# A Precision Microbiome Approach Using Sucrose for Selective Augmentation of *Staphylococcus epidermidis* Fermentation against *Propionibacterium acnes*

**DOI:** 10.3390/ijms17111870

**Published:** 2016-11-09

**Authors:** Yanhan Wang, Ming-Shan Kao, Jinghua Yu, Stephen Huang, Shinta Marito, Richard L. Gallo, Chun-Ming Huang

**Affiliations:** 1 Department of Dermatology, School of Medicine, University of California, San Diego, CA 92093, USA; yanhanw@gmail.com (Y.W.); rgallo@ucsd.edu (R.L.G.); 2 Department of Biomedical Sciences and Engineering, National Central University, Taoyuan 320009, Taiwan; s36424592@yahoo.com.tw (M.-S.K.); shintasimbolon53@yahoo.com (S.M.); 3 NMR and Crystallography Facilities, Sanford-Burnham Institute for Medical Research, La Jolla, CA 92037, USA; jinghua@sbpdiscovery.org; 4 Surface Bioadvances Inc., San Diego, CA 92121, USA; rhuang53@gmail.com; 5 Moores Cancer Center, University of California, San Diego, CA 92103, USA

**Keywords:** acne vulgaris, microbiome, *P. acnes*, *S. epidermidis*, skin, sucrose

## Abstract

Acne dysbiosis happens when there is a microbial imbalance of the over-growth of *Propionibacterium acne*s (*P. acnes*) in the acne microbiome. In our previous study, we demonstrated that *Staphylococcus epidermidis* (*S. epidermidis*, a probiotic skin bacterium) can exploit glycerol fermentation to produce short-chain fatty acids (SCFAs) which have antimicrobial activities to suppress the growth of *P. acnes*. Unlike glycerol, sucrose is chosen here as a selective fermentation initiator (SFI) that can specifically intensify the fermentation activity of *S. epidermidis*, but not *P. acnes*. A co-culture of *P. acnes* and fermenting *S. epidermidis* in the presence of sucrose significantly led to a reduction in the growth of *P. acnes*. The reduction was abolished when *P. acnes* was co-cultured with non-fermenting *S. epidermidis*. Results from nuclear magnetic resonance (NMR) analysis revealed four SCFAs (acetic acid, butyric acid, lactic acid, and succinic acid) were detectable in the media of *S. epidermidis* sucrose fermentation. To validate the interference of *S. epidermidis* sucrose fermentation with *P. acnes*, mouse ears were injected with both *P. acnes* and *S. epidermidis* plus sucrose or phosphate buffered saline (PBS). The level of macrophage-inflammatory protein-2 (MIP-2) and the number of *P. acnes* in ears injected with two bacteria plus sucrose were considerably lower than those in ears injected with two bacteria plus PBS. Our results demonstrate a precision microbiome approach by using sucrose as a SFI for *S. epidermidis*, holding future potential as a novel modality to equilibrate dysbiotic acne.

## 1. Introduction

Microbial imbalance in the human skin microbiome [1,2] has been termed “skin dysbiosis” [3]. Here we refer to the precision microbiome as an approach for normalization of dysbiotic microbiome by selectively targeting specific microbes. *Propionibacterium acnes* (*P. acnes*) and *Staphylococcus epidermidis* (*S. epidermidis*) are two major bacterial inhabitants of lesions in acne vulgaris [2]. Although both *P. acnes* and *S. epidermidis* are human skin commensals, the over-growth of *P. acnes* has been recognized for its association with the progression of acne vulgaris [1,3]. The biological role of *S. epidermidis* within acne lesions remains mysterious. During the development of acne vulgaris, *S. epidermidis* may irregularly migrate from the outer surface of the skin into the hair follicle, where opportunistic strains of *P. acnes* exclusively habitate. A closed comedone (or a deep-seated abscess in an open comedone) creates an anaerobic microenvironment which may facilitate bacterial fermentation in an acne lesion. Results in our publication have demonstrated that *S. epidermidis* can ferment glycerol and create inhibition zones to repel a colony of over-grown *P. acnes* [4]. Both topical and intralesional application of a short-chain fatty acid (SCFA) produced by *S. epidermidis* glycerol fermentation into *P. acnes*-induced lesions markedly suppressed the bacterial colonization and inflammation in mice [4]. Although *S. epidermidis* is not well-defined as a probiotic bacterium, our previous results suggest that *S. epidermidis* can exploit glycerol fermentation against *P. acnes*.

Both *S. epidermidis* and *P. acnes* can fermentatively metabolize glycerol to produce SCFAs [4,5]. Although the oppositional relationship between *S. epidermidis* and *P. acnes* in human skin has not yet been clinically proven, we conjecture that, during the development of acne vulgaris, *S. epidermidis* and *P. acnes* may use the glycerol as a shared carbon source and produce different SCFAs as antimicrobial agents to compete against each other within an acne lesion. The acne vulgaris develops and persists when *P. acnes* becomes dominant during the bacterial interference. Our approach to treating acne vulgaris is to deter the over-growth of *P. acnes* by enhancing the fermentation activities of *S. epidermidis* by using a specific sugar substrate as a selective fermentation initiator (SFI).

It has been reported that different bacterial species express distinct enzymes that ferment specific sugar substrates [6,7]. Many bacteria use glucose, because they possess the enzymes required for the degradation and oxidation of this sugar. Fewer bacteria are able to use complex carbohydrates like disaccharides (lactose or sucrose) or polysaccharides (starch); these bacteria produce enzymes to hydrolyse glycosidic bonds of disaccharides. The monosaccharides from degraded disaccharides become new substrates for bacterial fermentation. *S. epidermidis*, but not *Staphylococcus aureus* (*S. aureus*), can ferment mannose and galactose [6,7]. Our previous results showed that both *S. epidermidis* and *P. acnes*, but not *S. aureus* (USA300), can fermentatively metabolize glycerol [8]. Although *S. aureus* bacteria utilize several carbohydrates as substrates for fermentation, they cannot ferment ducitol (also called galactitol, a sugar alcohol) or saccharic acid [7]. Unlike glucose and glycerol, which are endogenous metabolites in human skin, sucrose (C_12_H_22_O_11_)—a disaccharide combination of glucose and fructose sugars—is found naturally in many fruits and vegetables. It has been documented that sucrose is nonfermentable by *P. acnes*, and it has been used to reduce water activity and hence bacterial colonization of wounds [9].

Antibiotics without bacterial specificity for acne treatment may destroy the fermenting bacteria that help rein in the over-growth of *P. acnes* and maintain homeostasis of the acne microbiome. At dermatology clinics, acne cyst injections by direct injection of corticosteroids into the nodule can swiftly reduce redness and inflammation and help to immediately relieve the threat of scarring. However, this injection can cause local side effects, including pigmentary changes and atrophy [10]. Benzoyl peroxide—a topical medication for acne vulgaris—can cause severe skin irritation. Isotretinoin is a potent anti-acne agent derived from vitamin A [11]. However, it is strictly regulated because of its known adverse effects on birth defects. However, it is strictly regulated due to the induction of unwanted side effects. None of the above treatments selectively improve the fermentation activity of commensal skin bacteria that may have a lower risk of developing side effects and resistant *P. acnes*. Unlike vaccines, the action of probiotics does not directly require host immune stimulation, and may have little or no disruption to other commensal bacteria. However, live *S. epidermidis* as probiotics, when added exogenously into the open lesion/comedone of acne vulgaris, may be not a viable option in patients with significant underlying health issues, such as surgical intervention and immune suppression. In this study, we employ a precision microbiome approach by selective augmentation of the fermentation activities of *S. epidermidis* against *P. acnes*. The development of sucrose as a targeted intervention specifically for *S. epidermidis* fermentation may be relatively safe when it is employed to rebalance dysbiotic acne.

## 2. Results

### 2.1. Sucrose Selectively Triggered S. epidermidis, but Not P. acnes, to Undergo Fermentation

**Figure 1:**
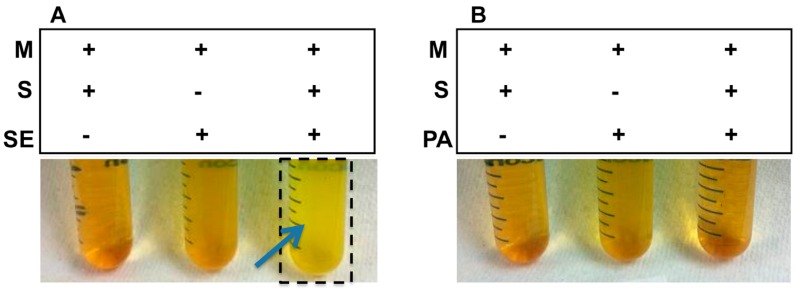
Sucrose as a selective carbon source for *Staphylococcus epidermidis* (*S. epidermidis*) fermentation. (**A**) *S. epidermidis* (SE) or (**B**) *Propionibacterium acnes* (*P. acnes*, PA) (10^5^ CFU/mL) was incubated in rich media (M) with or without 20 g/L sucrose (S) for six days. Rich media plus sucrose without bacteria was included as a control. *S. epidermidis*, but not *P. acnes*, fermented sucrose. A color change to yellow in the media (marked in a black frame and blue arrow) indicates that sucrose fermentation of *S. epidermidis* has occurred. Representative data from three independent experiments are shown.

To examine the sucrose fermentation activities of *S. epidermidis* and *P. acnes*, bacteria were incubated in rich medium under anaerobic conditions in the presence of 20 g/L sucrose. Rich media plus either sucrose or bacteria were used as controls. To monitor the fermentation process, phenol red (a fermentation indicator) was added into the culture to assess SCFA production as a result of sucrose fermentation. Only media in the culture of *S. epidermidis* (ATCC 12228), but not *P. acnes* (ATCC 6919), with sucrose turned yellow (more acidic) after six days of incubation (Figure 1), indicating that sucrose can selectively trigger *S. epidermidis* to undergo fermentation.

### 2.2. The Sucrose Fermentation of S. epidermidis Is Essential for Inhibition of P. acnes Growth

**Figure 2:**
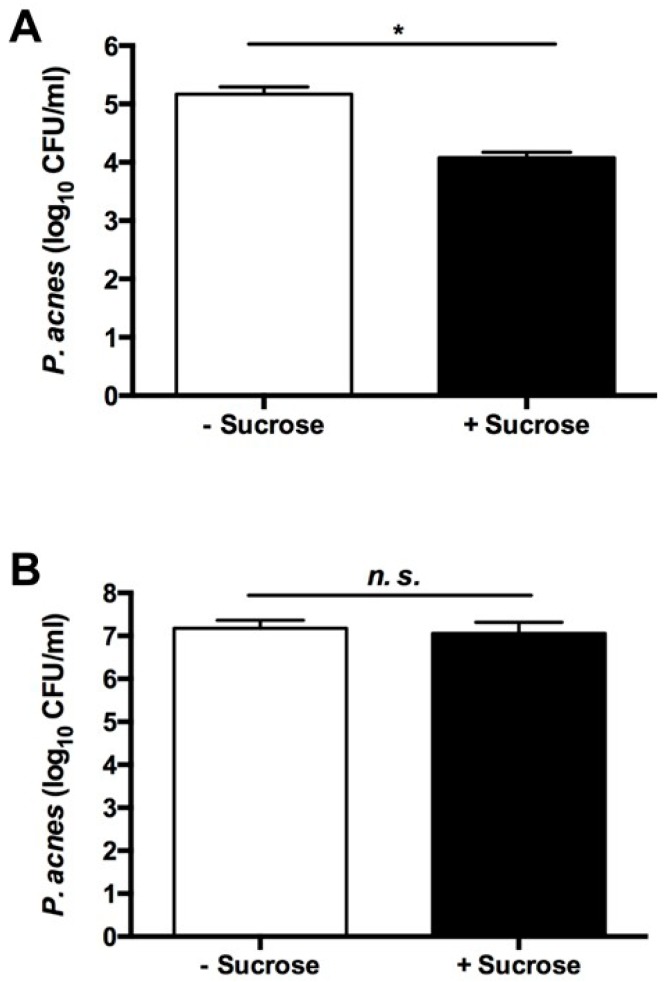
The essential role of sucrose fermentation of *S. epidermidis* in the inhibition of the growth of *P. acnes*. (**A**) Fermenting *S. epidermidis* (ATCC 12228) or (**B**) non-fermenting *S. epidermidis* (10^6^ CFU) were co-incubated with *P. acnes* (10^6^ CFU) in 10 mL rich media with or without 20 g/L sucrose for three days. After incubation, culture media containing bacteria were diluted 1:10–1:10^5^ with phosphate buffered saline (PBS) and then spotted the dilution (5 μL) onto *P. acnes* selective agar plates which contain rich media and 10 μg/mL of furazolidone. The number of *P. acnes* six days after incubation is expressed as log_10_ CFU/mL. Data are the mean ± standard deviation (SD) of three separate experiments. * *p* < 0.05 (two-tailed *t*-tests). *n.s*. = not significant.

To examine whether the sucrose fermentation of *S. epidermidis* hindered the growth of *P. acnes*, *S. epidermidis* and *P. acnes* were co-cultured in the presence or absence of sucrose. To establish a *P. acnes*-selective plate, media from the co-culture of *S. epidermidis* and *P. acnes* were spotted on a rich medium plate supplemented 10 μg/mL of furazolidone (Furoxone). We found that furazolidone at a concentration of 10 µg/mL can completely kill *S. epidermidis* without affecting the growth of *P. acnes* (data not shown). Three days after the co-culture of *S. epidermidis* and *P. acnes* with/without sucrose, media were spotted on a *P. acnes*-selective plate. After co-culture of *S. epidermidis*/*P. acnes* in the absence of sucrose, *P. acnes* formed 1.4 ± 0.29 × 10^5^ CFUs on a plate. However, when sucrose was present in the co-culture, an approximately one log_10_ reduction in the number of *P. acnes* colonies (1.2 ± 0.01 × 10^4^ CFUs) was observed (Figure 2A). These findings suggest that *S. epidermidis* mediated sucrose fermentation to interfere with the growth of *P. acnes*.

To validate the essential role of *S. epidermidis* fermentation in the interference with the growth of *P. acnes*, we screened the fermentation activities of different *S. epidermidis* strains isolated from human fingertips. In the presence of sucrose, the media in the culture of a strain with a 16S rRNA sequence showing 99% identity to that in *S. epidermidis* ATCC 12228 [12] remained orange-red after six days of incubation (Figure S1). We thus define this strain as a non-fermenting *S. epidermidis*. When a co-culture of non-fermenting *S. epidermidis* with *P. acnes* was conducted in the presence of sucrose, no reduction in the number of *P. acnes* colonies was found (Figure 2B), suggesting that fermentation is required for *S. epidermidis* to restrain the growth of *P. acnes*.

### 2.3. Identified SCFAs in Fermented Media of S. epidermidis

**Figure 3:**
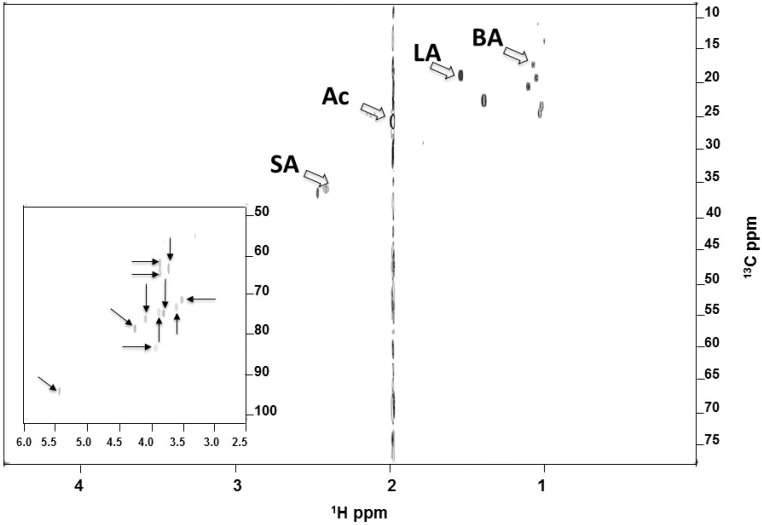
Short-chain fatty acid (SCFA) identification by NMR analysis. The media of *S. epidermidis* ^13^C_12_-sucrose fermentation were centrifuged and passed through a 0.2 μm filter. Supernatants were then mixed with 10% D_2_O and analyzed by NMR spectrometers. A 2-D ^1^H-^13^C HSQC NMR spectrum (600 MHz) was displayed. The un-metabolized ^13^C_12_-sucrose (solid arrows) appears 2.5–6.0 and 50–100 ppm in the ^1^H- and ^13^C-NMR spectra, respectively. Besides sucrose, four SCFAs (acetic (Ac), butyric (BA), lactic (LA), and succinic acids (SA), open arrows) were detected in the ferments of *S. epidermidis*.

To identify the SCFAs during fermentation, ^13^C_12_-sucrose (20 g/L) was added into the culture of *S. epidermidis* (ATCC 12228) under anaerobic conditions for six days. Supernatants of bacterial culture were mixed with 10% deuterium oxide (D_2_O) for one-dimensional (1-D) (data not shown) and two-dimensional (2-D) (Figure 3) ^13^C and ^1^H nuclear magnetic resonance (NMR) analysis. Besides un-metabolized sucrose, four SCFAs (acetic, butyric, lactic, and succinic acids) were detected in the fermented media of *S. epidermidis*, demonstrating the bacterial capability of fermentatively metabolizing sucrose into SCFAs. These results demonstrate that *S. epidermidis* fermentatively metabolized ^13^C_12_-sucrose into SCFAs. Results in our previous publication have demonstrated that SCFAs can suppress the growth of *P. acnes* ATCC 6919 [4]. To examine if SCFAs have the ability to kill *P. acnes* strains isolated from acne lesions, the minimum bactericidal concentration (MBC) value of acetic acid for a *P. acnes* strain isolated from acne lesion was determined (Figure S2). *P. acnes* was incubated with varying concentrations (0, 5, 7.5, 10, 25 and 50 mM) of acetic acid overnight at 37 °C. After incubation, bacteria diluted with PBS were spotted on an agar plate for counting the colony forming units (CFU). Acetic acid reduced the *P. acnes* growth by more than one log_10_ at a concentration greater than 7.5 mM, and completely killed *P. acnes* at a concentration higher than 25 mM. These results suggest that SCFAs produced by sucrose fermentation of *S. epidermidis* may be able to impede the growth of *P. acnes* in acne vulgaris.

### 2.4. S. epidermidis Sucrose Fermentation Abrogated P. acnes-Induced Inflammation and Bacteria Colonization In Vivo

**Figure 4:**
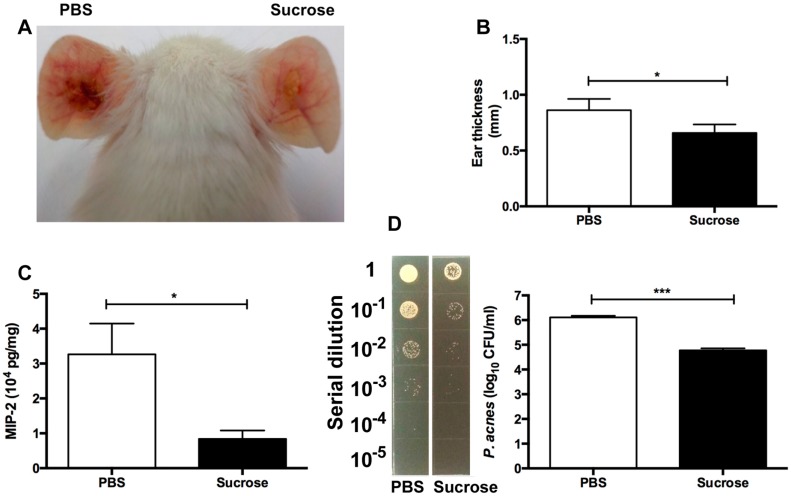
In Vivo reduction of *P. acnes* colonization and inflammation by *S. epidermidis* sucrose fermentation. The ears of Institute for Cancer Research (ICR) mice were injected intradermally with *P. acnes* (ATCC 6919) (10^7^ CFU) and *S. epidermidis* (ATCC 12228) (10^7^ CFU) with sucrose (20 g/L in 10 μL PBS) or 10 μL PBS. (**A**) A photo of ear inflammation was taken three days after injection; (**B**) The ear thickness (mm); (**C**) The levels of macrophage-inflammatory protein-2 (MIP-2) cytokines in the ears injected with bacteria in the presence of sucrose or PBS was measured by an enzyme-linked immunosorbent assay (ELISA) kit; (**D**) The CFUs in the ears injected with bacteria in the presence of sucrose or PBS were enumerated by plating serial dilutions (1:10–1:10^5^) of the homogenate on an agar plate. Three days after injection, *p*-values were evaluated using two-tailed *t*-tests. Data are the means of three separate experiments using four mice per group. *** *p* < 0.001; * *p* < 0.05.

To test whether *S. epidermidis* can counteract *P. acnes* in the presence of sucrose, the ears of Institute for Cancer Research (ICR) mice were injected intradermally with *P. acnes* (10^7^ CFU) and *S. epidermidis* (10^7^ CFU) in the presence of sucrose (20 g/L) or PBS for three days. As shown in Figure 4, compared to those in the mice injected with two bacteria and PBS, both ear redness and thickness in the mice injected with two bacteria and sucrose was significantly lower (Figure 4A,B). To determine whether *S. epidermidis* sucrose fermentation can ameliorate the production of *P. acnes*-induced pro-inflammatory cytokines, mouse ears were excised and homogenized three days after injection. The level of macrophage-inflammatory protein-2 (MIP-2)—a murine counterpart of human interleukin (IL) 8—was measured by an enzyme-linked immunosorbent assay (ELISA). MIP-2 production in the ear injected with two bacteria and sucrose was approximately 75% (solid bar; 0.84 ± 0.17 × 10^4^ pg/mL) less than that detected in the ear injected with two bacteria and PBS (open bar; 3.26 ± 0.51 × 10^4^ pg/mL) (Figure 4C).

To explore if *S. epidermidis* sucrose fermentation can hinder the growth of *P. acnes*, the ears of mice injected with two bacteria along with sucrose or phosphate buffered saline (PBS) were excised and homogenized. As shown in Figure 4D, the number of *P. acnes* recovered from mouse ears administered with two bacteria plus sucrose (solid bar; 6.0 ± 0.07 × 10^4^ CFUs) was much lower than that recovered from ears administered with two bacteria plus PBS (open bar; 12.9 ± 1.1 ×10^5^ CFUs), suggesting the suppression of *P. acnes* growth by *S. epidermidis* sucrose fermentation.

## 3. Discussion

Sugars hold inherent antibacterial properties, some of which are due to high osmotic pressure and low water activity, which is inhibitory to the growth of bacteria [13]. It has been reported that the inhibitory effect of sucrose against *S. aureus* was primarily attributed to its water activity-lowering ability [14]. To examine the possible antibacterial activity of sucrose, we incubated *P. acnes* or *S. epidermidis* with 2% (20 g/L) sucrose overnight. As shown in Figure S3, incubation of 2% sucrose did not affect the growth of *P. acnes* or *S. epidermidis*. The result suggests that a decrease in the number of *P. acnes* in the co-culture of *S. epidermidis* and *P. acnes* with 2% sucrose (Figure 2A) primarily resulted from *S. epidermidis* sucrose fermentation, not antibacterial property of sucrose. As shown in Figure 3, four SCFAs were produced in the fermented media of *S. epidermidis*. Results in our previous papers demonstrated that pH buffering does not influence the antimicrobial activities of SCFAs [15]. Furthermore, SCFAs can passively diffuse through the cell wall of bacteria and kill bacteria by lowering the intracellular pH [8]. Findings above suggested that the suppression of *P. acnes* growth in a co-culture assay (Figure 2) and by acetic acid (Figure S2) was not simply due to the acidities of SCFAs in media.

By employing an approach of precision programming of the acne microbiome, we use sucrose as a SFI for *S. epidermidis* in this study. Phage therapy is a precision microbiome approach wherein scientists use bacteriophages specifically targeting pathogens without damaging the commensal bacteria of the host [16]. Although bacteriophages can be highly bacteria-specific, many disadvantages of phage therapy have been documented [17]. For example, for the selection of bacteriophages as potential safe antimicrobials, detailed comprehensive characteristics of genome and phenotypic properties may be required, since bacteriophages contain negative features such as lysogeny-associated genes, toxin or enzyme encoding genes [18]. Furthermore, scientists do not yet fully appreciate the safety issue of phage therapy, because many identified genes of bacteriophages express hypothetical and putative proteins with predicted or unknown functions. An additional drawback of phage therapy is that bacteriophages can transfer their genes from one microbe to another. Sucrose—a substance of extremely low acute toxicity—is Generally Recognized As Safe (GRAS) by the US Food and Drug Administration (FDA). With hygroscopic properties, sucrose is commonly used in hair and skin care products to retain moisture. Although sucrose is used here as a SFI for *S. epidermidis*, future studies will include determining whether sucrose influences the growth and fermentation activity of other commensal bacteria—especially *S. aureus*—in skin.

Genetic loci responsible for sucrose fermentation of *S. epidermidis* have not been described. Several enzymes (e.g., permease) regulate the movement of carbohydrates across the bacterial cell wall [19]. We believe that sucrose fermentation, an anaerobic process, also requires many intracellular enzymes that degrade sucrose to SCFAs. Once the key enzyme in charge of sucrose fermentation is determined, a mutant strain of *S. epidermidis* with a deficiency in this enzyme can be created to validate the essential role of fermentation in the suppression of *P. acnes* growth. Low levels of SCFAs in the human bloodstream were detected, ranging from 3 to 7 µM [20]. However, the intestinal microbes in the human colon can locally produce high levels (20–140 mM) of SCFAs [20] that can sufficiently kill pathogens. Although we have not determined how many SCFAs can be locally produced when sucrose is applied to the microenvironment of an acne lesion, it has been documented that SCFAs with short half-lives have apparent difficulty achieving pharmacologic concentrations In Vivo [21]. Several pro-drugs of butyric acid (e.g., pivaloylomethyl butyrate; AN-9) [21] have been developed to achieve effective concentrations of butyric acid. SCFAs act on host cells through at least two mechanisms: inhibition of histone deacetylase (HDAC) and activation of free fatty acid receptors (Ffar1; also known as G-protein coupled receptor 41 (GPR41) and Ffar2; GPR43) [22]. The expression of Ffar1 [23] and Ffar2 [24] in acne lesions is not yet quantified. Several HDAC inhibitors with anti-inflammatory activities are in preclinical and clinical development, including AN-9. Butyric acid is a small molecular weight carboxylate that is a class I HDAC inhibitor [25]. Propionic and butyric acids have been verified as ligands of Ffar1 [26]. Previous studies showed that GW9508 (GlaxoSmithKline)—an arylalkyl derivative of propionic acid—activated the Ffar1 receptor, suppressed chemokine induction in keratinocytes, and attenuated cutaneous immune inflammation [27]. *P. acnes* can activate toll-like receptor 2 (TLR-2) [28] to stimulate the secretion of IL-6 and IL-8 by follicular keratinocytes, and IL-1β, tumor necrosis factor alpha (TNFα), IL-8, and IL-12 by monocytic cells [29]. As shown in Figure 4C, we demonstrated a reduction of MIP-2 cytokine when mouse ear was injected with *P. acnes* and *S. epidermidis* in the presence of sucrose. It is possible that SCFAs produced by *S. epidermidis* attenuate *P. acnes-*induced inflammation via the inhibition of HDACs or the activation of Ffar1 or Ffar2. Our previous studies have demonstrated that a co-drug—butyric acid 2-(2-butyryloxyethoxy) ethyl ester—can release two active butyric acids in mice (data not shown). A co-drug—propionic acid 2-(2-propionyloxy-ethoxy)-ethyl ester—exerts antimicrobial activity [15]. Application of these co-drugs onto acne lesions may conquer the drawbacks of SCFAs with short half-lives for acne treatments.

Our results in Figure 4 show that intradermal injection of *P. acnes* into mouse ears provoked a significant granulomatous response, which was characterized as a lesion of epithelioid macrophages, frequently bounded by a lymphocyte cuff [30]. In the event of severe acne vulgaris, *P. acnes* could move into the dermal layer once the follicular wall was ruptured [31]. Injection of *P. acnes* into mouse ears may represent an animal model for the granulomatous type of inflammatory acne vulgaris that follows follicular rupture. *P. acnes* has been subdivided into at least two types (I and II), and two subtypes, IA and IB [32]. A recent study demonstrated that an acne lesion in humans consists of mixed *P. acnes* phenotypes [33]. Although we do not know which subtypes of *P. acnes* we isolated from acne lesions (Figure S2), the development of SCFA co-drugs that can specifically suppress the growth of acne-associated *P. acnes* subtypes may be necessary to avoid the disruption of the homeostasis of commensal *P. acnes* subtypes. We also do not know whether all strains of *S. epidermidis* in acne lesions have equal abilities to ferment sucrose. It has been reported that IL-8 was expressed at a higher level in acne lesions than that in healthy skin [34]. As shown in Figure S4A, the level of IL-8 cytokine was significantly reduced when an acne lesion was incubated with (20 g/L) sucrose for 24 h. *P. acnes* bacteria in acne lesions were detected by immunohistochemical staining with a monoclonal antibody against a surface sialidase protein of *P. acnes*. Incubation of sucrose significantly lowered the numbers of *P. acnes* in acne lesions (Figure S4B–F). A clinical study with additional acne lesions to determine the production of SCFAs by sucrose fermentation and the efficacy of sucrose as a SFI for *S. epidermidis* against *P. acnes* in acne lesions may be required in the future.

Clindamycin and erythromycin are commonly-prescribed topical antibiotics for acne vulgaris [35]. Antibiotic single-agent therapy can result in rapid development of clinically significant antibiotic resistance [36]. Several sugars have been added into antibiotic formula as ingredients. For example, sucrose is often added into amoxicillin for formulation [37]. An adjuvant is a pharmacological or immunological agent that modifies the effect of a drug or vaccine. Formulation of an antibiotic with sucrose which functions as an adjuvant may potentiate the effect of the antibiotic on killing of *P. acnes*. The development of sucrose as post-antibiotic adjuvant therapy [38] may lower the required dose of antibiotic for the treatment of acne vulgaris, decreasing the risk of generating resistant *P. acnes* and non-specific killing effect of antibiotics on skin commensal bacteria.

## 4. Experimental Section

### 4.1. Ethics Statement

Experiments using mice were performed at National Central University (NCU). The NCU ethics committee specifically approved this study (ID: 104-2320-B-008-003) on August 01, 2015 under an approved Institutional Animal Care and Use Committee (IACUC) protocol.

### 4.2. Bacterial Culture

*S. epidermidis* bacteria, including ATCC 12228 and non-fermenting *S. epidermidis* isolated from human skin, were cultured on 3% tryptic soy broth (TSB) (Sigma, St. Louis, MO, USA) agar plates overnight at 37 °C. *P. acnes* bacteria (ATCC 6919) were cultured on Brucella broth agar plates, supplemented with 5% (*v*/*v*) vitamin K (Remel, Lenexa, KS, USA) and hemin (Remel) under anaerobic conditions using Gas-Pak (BD Biosciences, San Jose, CA, USA) at 37 °C with shaking at 200 rpm. A single colony was inoculated in 3% TSB and Reinforced Clostridium Medium (Oxford, UK) and cultured at 37 °C until the logarithmic growth phase. Bacterial pellets were harvested by centrifugation at 5000× *g* for 10 min, washed with PBS, and suspended in PBS.

### 4.3. Fermentation of Bacteria

*S. epidermidis* and *P. acnes* bacteria (10^5^ CFU/mL) were incubated in 10 mL rich media (10 g/L yeast extract (Biokar Diagnostics, Beauvais, France), 3 g/L TSB, 2.5 g/L K_2_HPO_4_, and 1.5 g/L KH_2_PO_4_) in the absence and presence of 20 g/L sucrose under anaerobic conditions at 37 °C with shaking at 200 rpm. The rich media plus 20 g/L sucrose without bacteria were included as a control. The 0.002% (*w*/*v*) phenol red (Sigma, St. Louis, MO, USA) in rich media with 20 g/L sucrose acted as a fermentation indicator. A color change from red-orange to yellow indicated the occurrence of bacterial fermentation.

### 4.4. Co-Culture Assays

*S. epidermidis* (10^6^ CFU) and *P. acnes* (10^6^ CFU) bacteria were co-incubated in 10 mL rich media with or without 20 g/L sucrose under anaerobic conditions for three days at 37 °C with shaking at 200 rpm. After incubation, bacteria were diluted 1:10–1:10^5^ with PBS and then spotted the dilution (5 μL) onto *P. acnes* selective agar plates containing rich media and 10 μg/mL of furazolidone (Sigma) [39]. The CFUs of *P. acnes* were counted after incubation at 37 °C for six days.

### 4.5. NMR Analysis

*S. epidermidis* (ATCC 12228) (10^5^ CFU/mL) bacteria were incubated in rich media in the presence of ^13^C_12_-sucrose (20 g/L) (Omicron Biochemicals, Inc., South Bend, USA) for six days. The 1-D NMR spectra of SCFAs were obtained via a 400 MHz JEOL-ECS NMR spectrometer. The 2-D ^1^H-^13^C heteronuclear single quantum correlation (HSQC) spectra of SCFAs were acquired on a Bruker Avance 600 MHz NMR spectrometer with a triple resonance inverse (TCI) cryo-probe. The 2048 × 256 complex data points were recorded with 32 scans and a repetition time of 1 s, as previously described in detail [4]. Newly appearing peaks are the intermediate or final products produced by ^13^C_12_-sucrose fermentation of *S. epidermidis*.

### 4.6. The Sucrose Fermentation of S. epidermidis against P. acnes In Vivo

The ICR mice (2–3-month-old females; Harlan Labs, Placentia, CA, USA) were anesthetized by isoflurane (Sigma). Five mice per group were used in each experiment. The ears of ICR mice were injected intradermally with *P. acnes* (ATCC 6919) (10^7^ CFU) and *S. epidermidis* (ATCC 12228) (10^7^ CFU) with sucrose (20 g/L in 10 μL PBS) or 10 μL PBS using an insulin syringe with 29 G × 1/2 inches (BD Biosciences, San Jose, CA, USA). After three days, ear thickness was measured with an electronic digital caliper (Mitutoyo, Kanagawa, Japan). Ears were excised, weighed, and homogenized for cytokine detection and bacterial counts. The total protein concentration was measured by a Pierce BCA Protein Assay Kit (Thermo Scientific, Waltham, MA, USA).

### 4.7. Bacterial Loads in Mouse Ears

After excising mouse ears, tissue homogenates were made by a tissue grinder in 200 μL of sterile PBS. CFUs of *P. acnes* in ear homogenates were enumerated by plating serial dilutions (1:10–1:10^5^) of homogenates on *P. acnes* selective agar plates containing rich media and 10 μg/mL of furazolidone (Sigma). After that, plates were incubated for three days at 37 °C under anaerobic conditions using Gas-Pak. A biosafety level 2 (BSL-2) facility was used for conduction of mouse experiments in accordance with institutional guidelines for animal experiments.

### 4.8. ELISA

The MIP-2 in the supernatants of ear homogenates was quantified by an ELISA kit, as directed by the manufacturer (R&D System. Inc., Minneapolis, MN, USA).

### 4.9. Statistics

Experiments were repeated at least three times with similar results. Statistical significance was determined using Student’s unpaired two-tailed *t*-test, as indicated in the legend (* *p* < 0.05, ** *p* < 0.01, *** *p* < 0.001).

## 5. Conclusions

Overall, in this study, sucrose was able to specifically intensify the probiotic ability of *S. epidermidis*, producing SCFAs which can potentially suppress the inflammation and growth of *P. acnes*. The novel acne treatment using sucrose as a SFI for probiotic *S. epidermidis* may benefit the entire community of patients with acne vulgaris, consisting of over 50,000 patients in the US [40].

## Supplementary Materials

Supplementary materials can be found at www.mdpi.com/1422-0067/17/11/1870/s1.

## Abbreviations

Ac	acetic acid
ATCC	American Type Culture Collection
BA	butyric acid
CFU	colony-forming unit
BSL-2	biosafety level 2
1-D	one-dimensional
2-D	two-dimensional
D_2_O	deuterium oxide
ELISA	enzyme-linked immunosorbent assay
FDA	Food and Drug Administration
Ffar1	free fatty acid receptor 1
Ffar2	free fatty acid receptor 2
GPR41	G-protein coupled receptor 41
GRAS	Generally Recognized As Safe
HDAC	histone deacetylase
HSQC	heteronuclear single quantum coherence
IACUC	Institutional Animal Care and Use Committee
IL	interleukin
MIP-2	macrophage-inflammatory protein-2
NCU	National Central University
NIH	National Institutes of Health
NF-*S. epidermidis*	non-fermentation *S. epidermidis*
NMR	nuclear magnetic resonance
NS	non-significant
*P. acnes*	*Propionibacterium acnes*
PBS	phosphate buffered saline
SCFA	short-chain fatty acids
SD	standard deviation
*S. epidermidis*	*Staphylococcus epidermidis*
SFI	selective fermentation initiator
16S rRNA	16S ribosomal RNA
TLR-2	toll-like receptor 2
TNFα	tumor necrosis factor α
