# Supplementary material for: A Precision Microbiome Approach Using Sucrose for Selective Augmentation of Staphylococcus epidermidis Fermentation against Propionibacterium acnes
Source: Int J Mol Sci. 2016 Nov 9;17(11):1870. doi: 10.3390/ijms17111870 (PMC5133870; doi:10.3390/ijms17111870)
Supplement: Supplementary file 1 [file ijms-17-01870-s001.pdf]

Supplementary Material: A Precision Microbiome Approach Using Sucrose for Selective Augmentation of *Staphylococcus epidermidis* Fermentation against *Propionibacterium acnes*

Yanhan Wang, Ming-Shan Kao, Jinghua Yu, Stephen Huang, Shinta Marito, Richard L. Gallo and Chun-Ming Huang

Experimental Section

The MBC Assays

To determine the minimum bactericidal concentration (MBC) value of acetic acid, *P. acnes* bacteria isolated from acne lesions (10^7 CFU) were incubated with acetic acid at various concentrations (5–50 mM in phosphate buffered saline, PBS) in media on a 96-well microplate (100 μL per well) overnight. The control received only PBS. After incubation, bacteria were diluted 1:10–1:10^5 with PBS. MBC was finally examined at a 99.9% killing level and determined by spotting the dilution (5 μL) on an agar plate supplemented with media for the counting of CFUs.

Incubation of Acne Biopsies with Sucrose

The lesions (4 × 4 × 8 mm) of six patients with acne vulgaris within three to seven days of the onset of inflammatory papules were obtained by punch biopsy from back skins. The Institutional Review Board (IRB) at University of California, San Diego (UCSD) approved the consent procedure and sampling of acne biopsies under an approved protocol (no. 121230). The written consents from all participants were obtained before conducting sampling of acne biopsies. The lesion was cut in half, and half was incubated with 20 g/L sucrose in the antibiotic-free keratinocyte growth medium (Thermo Fisher Scientific) for 24 h. The other half was incubated in media without adding sucrose. The IL-8 in the supernatants of homogenates of acne lesions was depicted as pg/mg tissue (acne lesion) after quantification by ELISA using a Quantikine human IL-8 set (R&D System, Minneapolis, MN, USA). For counting *P. acnes*, acne lesions incubated with/without 20 g/L sucrose were embedded in Tissue-Tek O.C.T. (Sakura Finetek, Torrance, CA, USA) and subjected to immunohistochemical staining using a mouse monoclonal antibody against a surface sialidase (accession number: gi/50843035) of *P. acnes* and Alexa Fluor® 594 donkey anti-mouse IgG (H/L). Diamidino-2-phenylindole (DAPI) was used as a nuclear counterstain. Fluorescence was detected using a Bx51 research microscope (Olympus, Melville, NY, USA) in tandem with X-Cite 120 fluorescence illumination systems (EXFO, Ville de Québec, QC, Canada). The percentage of sialidase-positive stains indicated as *P. acnes* bacteria in acne lesions were calculated from approximately 65 DAPI-stained cells in a microscopic field. Three fields per acne lesion from three patients were randomly chosen for calculation.

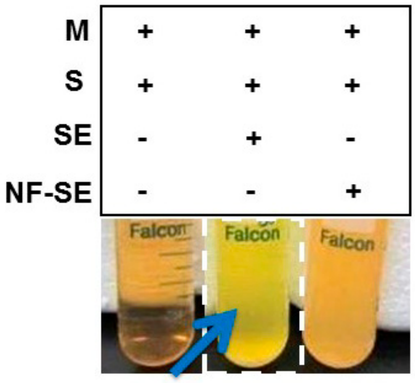

Figure S1. A sucrose non-fermenting *S. epidermidis* strain. The fermenting *S. epidermidis* (ATCC 12228) (SE) (Figure 1A) or a non-fermenting *S. epidermidis* strain (NF-SE) (10^5 CFU/mL) was incubated in phenol red-containing rich media (M) in the presence of sucrose (S) (20 g/L in 10mL) for six days under anaerobic conditions. Rich media plus sucrose without bacteria (the first tube on the left) were included as a control. Noticeable changes in color from orange-red to yellow were detected in the culture media of *S. epidermidis*, but not NF-*S. epidermidis*, validating that NF-*S. epidermidis* is a sucrose non-fermenting *S. epidermidis* strain. Results are representative of three separate experiments.

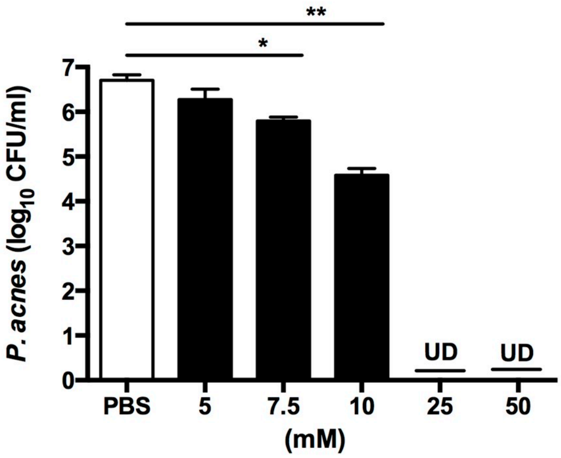

Figure S2. Anti-*P. acnes* activities of acetic acid. For minimum bactericidal concentration (MBC) assays, a *P. acnes* strain (10^7 CFU/mL) isolated from acne lesions was incubated with acetic acid (5–50 mM in PBS) or PBS alone on a 96-well microplate overnight. After incubation, *P. acnes* was diluted 1:10–1:10^5 with PBS, and 5 μL of the dilutions were spotted on an agar plate for CFU counts. The number of viable *P. acnes* is expressed as $\log_{10}$ CFU/mL. * $p < 0.05$; ** $p < 0.01$ (two-tailed *t*-tests). Data are the mean ± SD of three separate experiments. UD: undetectable.

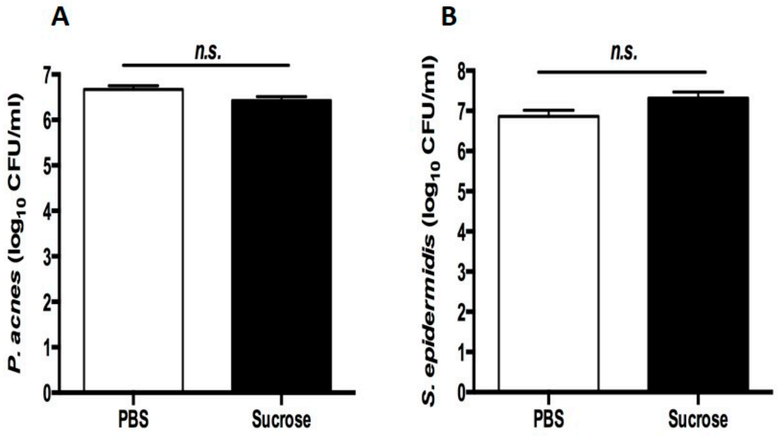

Figure S3. The effect of sucrose on the growth of *P. acnes* and *S. epidermidis*. *P. acnes* (ATCC 6919) or *S. epidermidis* (ATCC 12228) (10^7 CFU/mL) was incubated with 20 g/L sucrose or PBS on a 96-well microplate overnight. After incubation, bacteria were diluted 1:10–1:10^5 with PBS, and 5 μL of the dilutions were spotted on an agar plate for CFU counts. The number of viable (**A**) *P. acnes* or (**B**) *S. epidermidis* is expressed as $\log_{10}$ CFU/mL. Data are the mean ± SD of three separate experiments. n.s. = not significant.

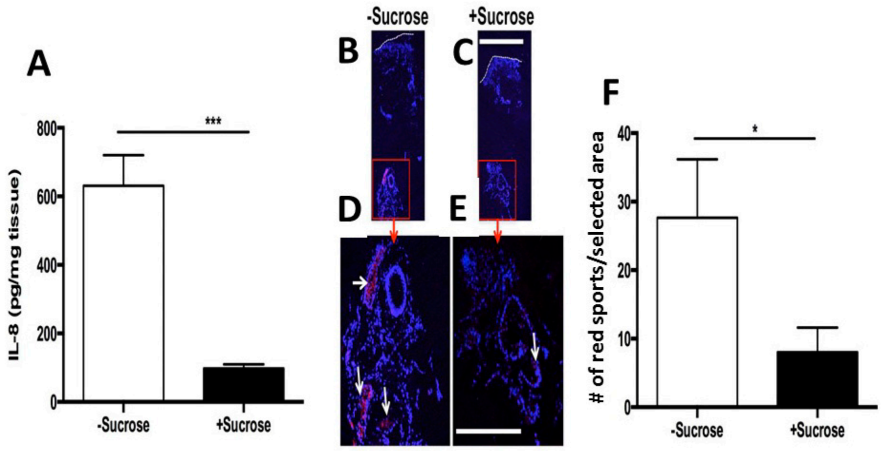

Figure S4. Incubation of acne lesions with sucrose decreases the level of IL-8 and the number of sialidase-stained *P. acnes*. (**A**) The acne lesions were obtained from back skin of three patients with acne lesions. Each lesion was cut in half and incubated with or without 20 g/L sucrose for 24 h. The levels of IL-8 cytokines in the supernatants of homogenates of acne lesions were quantified by ELISA assays. Immunohistochemical staining showed that the numbers of *P. acnes* bacteria (red arrows) in acne lesions incubated with (**C**,**E**) sucrose detected by a monoclonal antibody to sialidase of *P. acnes* (white arrows) were considerably lower than those in acne lesions incubated without (**B**,**D**). High resolution images from selected areas of (**C**,**B**) are shown in (**D**,**E**), respectively. DAPI (blue) labeled nuclei. Bars = 150 μm (**C**); 200 μm (**D**). Data are the mean ± SD of experiments using three acne lesions for IL-8 detection and three acne lesions for *P. acnes* counts. * p <0.05; *** $p < 0.001$ via two-tailed t-tests.
